# Montmorillonite–fungal biomass hybrid as sustainable sorbent in rotating-disk sorptive extraction coupled to GC–MS for trace-level monitoring of estrogens in hospital wastewater

**DOI:** 10.1007/s00216-026-06715-8

**Published:** 2026-08-26

**Authors:** Maby M. Martínez-Garzón, Milton Rosero-Moreano, Edwin David Morales-Álvarez, Cesar Augusto Ossa-Jaramillo, Juan Sebastian Arcila-Henao, Sandra Montoya-Barreto

**Affiliations:** 1 Departamento de Química, Facultad de Ciencias Exactas y Naturales, Universidad de Caldas, Calle 65 # 26-10, Manizales, 170004 Caldas, Colombia; 2 Doctorado en Ciencias-Química, Facultad de Ciencias Exactas y Naturales, Universidad de Caldas, Calle 65 # 26-10, Manizales, 170004 Caldas, Colombia; 3 Centro de Bioinformática y Biología Computacional de Colombia - BIOS, Ecoparque los Yarumos, edificio BIOS, Manizales , 170002 Caldas, Colombia; 4 Centro de Desarrollo Tecnológico—Planta de Bioprocesos y Agroindustria, Departamento de Ingeniería, Universidad de Caldas, Calle 65 # 26-10, Manizales, 170004 Caldas, Colombia

**Keywords:** Rotating-disk sorptive extraction, Biohybrid sorbent, Montmorillonite–*Pleurotus ostreatus*, Steroidal estrogens, Hospital wastewater, Green analytical chemistry

## Abstract

In this study, we pioneered a bio–inorganic hybrid material by combining montmorillonite (MMT) with *Pleurotus ostreatus* mycelium. Moving beyond routine extraction phases, we focused on the preparation, physicochemical characterization, and application of this unique biohybrid for rotating-disk sorptive extraction (RDSE). The engineered material successfully blends the structured mesoporosity and high surface area of pristine clay with the dense, functionalized biopolymer network of fungal hyphae. By deconvoluting the sorptive mechanism, we found that estrogen retention is driven by a highly cooperative network of non-covalent forces. At a near-neutral wastewater pH (6.9–7.2), where the target steroids remain neutral, mass transfer is governed by localized hydrogen bonding with both clay edge sites (Si–OH, Al–OH) and cell-wall functionalities (NH_2_, –OH), supplemented by stabilizing cation–π interactions involving interlayer Ca^2+^ ions. When we applied this method to determine four estrogens (E1, E2, EE2, and E3) via gas chromatography–mass spectrometry (GC–MS) in complex hospital wastewater, our MMT–fungus sorbent significantly outperformed conventional primary–secondary amine phases. The protocol yielded low detection limits (1.1–9.1 ng L^−1^), quantitative recoveries (95–100%), and narrow intra-laboratory precision (%RSD ≤ 8.5%; Horwitz ratio [HorRat], 0.12–0.35). Authentic effluents revealed critical steroidal micropollutant levels between 195 and 853 ng L^−1^. Finally, multi-metric sustainability tools confirmed a highly favorable environmental profile (AGREEprep = 0.75, VIGI = 80, BAGI = 52.5), confirming that high-performance analytical chemistry can actively coexist with eco-friendly material engineering.

**Graphical abstract:**

**Figure:**
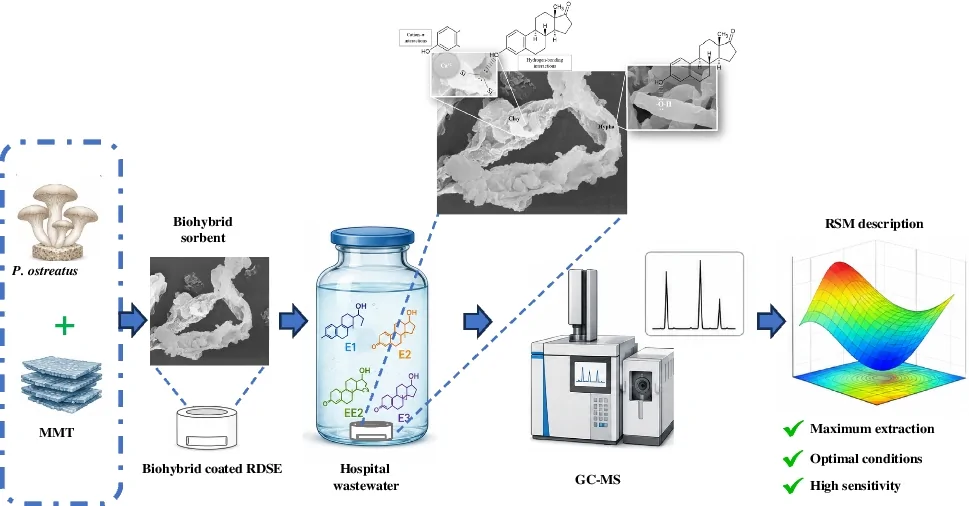

**Supplementary Information:**

The online version contains supplementary material available at https://doi.org/10.1007/s00216-026-06715-8.

## Introduction

Hospital facilities play a critical role in public health; however, their diverse medical units and services consume substantial volumes of water, generating considerable quantities of hospital wastewater (HWW). These effluents contain a complex, hard-to-treat mixture of hazardous substances—including pharmaceuticals, disinfectants, chemical reagents, detergents, and pathogenic microorganisms—that can severely affect both environmental and human health [1, 2]. According to data compiled by Majumder et al. [3], HWW generation ranges from 200 to 400 L per patient per day in high-income countries, and scales up to 400 to 1200 L in low- and middle-income nations, reflecting stark differences in hospital infrastructure, water consumption patterns, and wastewater management practices.

Among the various pollutants detected in HWW, emerging contaminants (ECs) have attracted considerable attention because of their persistence, bioaccumulation potential, and biological activity even at trace concentrations. Steroidal estrogens are particularly concerning endocrine-disrupting compounds (EDCs), capable of eliciting adverse physiological effects in aquatic life at concentrations in the low ng L^−1^ to sub-ng L^−1^ range [4–7]. The steroid hormones most frequently encountered in environmental matrices include estrone (E1), 17β-estradiol (E2), estriol (E3), and 17α-ethinylestradiol (EE2) [6]. We have summarized the main physicochemical properties and chemical structures of these hormones in Table S4.

The continuous release of steroidal estrogens from domestic, hospital, agricultural, and industrial sources has resulted in their widespread occurrence in aquatic environments. Once there, they can induce reproductive dysfunction, endocrine disruption, developmental abnormalities, and immunological alterations in aquatic organisms [6, 8]. Despite their environmental significance, comprehensive information regarding how these hormones behave and persist in hospital wastewater remains relatively scarce. Prior studies in Europe and Asia typically reported concentrations ranging from tens to several hundreds of nanograms per liter [9–12]. In contrast, recent monitoring campaigns conducted in a Colombian hospital wastewater treatment plant (HWWTP) revealed concentrations in the µg L^−1^ range—positioning them among the highest reported to date. Specifically, López-Vargas et al. [13], using rotating-disk sorptive extraction (RDSE) coupled with high-performance liquid chromatography with a photodiode array detector (HPLC-PDA) and ionic liquid-modified clay sorbents, reported influent concentrations of 4.30 µg L^−1^ (E3), 3.85 µg L^−1^ (E2), and 7.23 µg L^−1^ (EE2). Likewise, Martínez-Garzón et al. [14] measured alarming concentrations of 7.2 µg L^−1^ (E1), 0.1 µg L^−1^ (E2), 6.7 µg L^−1^ (EE2), and 33.2 µg L^−1^ (E3) within the same facility.

These findings underscore the urgent need for robust, sensitive, and reliable analytical methodologies to monitor steroidal estrogens in complex wastewater matrices. While the trace-level occurrence of these hormones has stimulated the development of various sample preparation techniques—such as solid-phase extraction (SPE), solid-phase microextraction (SPME), stir-bar sorptive extraction (SBSE), and RDSE [14–22]—several of these traditional approaches present clear practical limitations. Many current extraction phases require laborious synthesis procedures, display poor reusability, or rely on expensive commercial sorbents. Among these configurations, RDSE has emerged as a versatile and reusable microextraction alternative because it combines operational simplicity and low solvent consumption with the flexibility to incorporate custom-designed extraction phases. Consequently, developing sustainable and efficient sorbent materials has become a major driving force in sample preparation. Clay minerals such as montmorillonite (MMT) have attracted significant interest owing to their layered structure, large specific surface area, high cation-exchange capacity, and excellent adsorption properties [13, 23–25]. Concurrently, white-rot fungi such as *Pleurotus ostreatus* produce potent ligninolytic enzymes that can degrade a wide array of organic pollutants [26–28]. Beyond this enzymatic capability, their biomass is rich in diverse surface functional groups that readily interact with organic targets, making this species an excellent candidate for engineering advanced biohybrid sorbents [27, 29]. However, despite these distinct advantages, we noticed a clear gap in the literature: no studies have yet reported the preparation, characterization, and analytical application of a montmorillonite–*P. ostreatus* (MMT–fungus) biohybrid as an active extraction phase for RDSE. Furthermore, while our previous works demonstrated that RDSE can successfully determine steroidal estrogens using alternative sorbents [14, 30, 31], developing next-generation, eco-friendly extraction phases remains an open and vital research challenge.

To bridge this gap, we report the first preparation, comprehensive physicochemical characterization, and pioneering analytical application of an MMT–fungus biohybrid sorbent. We obtained this material via a one-pot biological strategy that integrates fungal biomass within a clay matrix, generating a heterogeneous hybrid architecture with interdependent mineral and biological domains. In contrast to previously reported clay-based sorbents, such as ionic liquid–intercalated MMT systems [13] or hydroxyapatite-modified MMT (MMT/HAp) composites with more homogeneous inorganic distributions [14], our proposed biohybrid exhibits a fundamentally different interfacial organization that enables multiple concurrent adsorption mechanisms. This unique structure successfully combines the natural adsorption capacity of MMT with the abundant functional groups of the fungal cell wall, creating a multifunctional surface where electrostatic interactions, hydrogen bonding, and surface complexation act synergistically. These features clearly distinguish our material from conventional RDSE sorbents, which typically rely on single-phase or chemically modified inorganic supports.

Finally, we successfully implemented this biohybrid as a reusable extraction phase in an RDSE–gas chromatography–mass spectrometry (GC–MS) workflow for the simultaneous determination of natural and synthetic steroidal estrogens in hospital wastewater. Crucially, the core novelty of this study lies in the development, characterization, and first analytical validation of this novel biohybrid material rather than modifications to the RDSE setup itself. With this approach, we aim to provide a highly sustainable, efficient, and cost-effective alternative for estrogen monitoring in complex aqueous matrices.

## Experimental section

### Materials and methods

#### Chemicals and materials

The analytical standards for estrone (E1), 17β-estradiol (E2), 17α-ethinylestradiol (EE2), and estriol (E3) were purchased from Sigma-Aldrich (St. Louis, MO, USA). For the extraction steps, we used HPLC-grade methanol (MeOH) and ethyl acetate, obtained from Merck (Darmstadt, Germany) and PanReac (Barcelona, Spain), respectively. To prepare the stock solutions, each individual hormone was dissolved in MeOH at 250 mg L^−1^, and we subsequently generated mixed working standards covering a concentration range of 0–100 µg L^−1^. For analyte derivatization, we selected *N,O*-bis(trimethylsilyl)trifluoroacetamide (BSTFA, HPLC grade) from Sigma-Aldrich as the silylation reagent, while the primary–secondary amine (PSA, *N*-propyl ethylenediamine) cleanup sorbent was supplied by Supelco (Bellefonte, PA, USA). High-purity helium and nitrogen gases (99.999%; Cryogas, Colombia) were used throughout the GC–MS analysis, where helium acted as the carrier gas and nitrogen was used to dry down the final extracts. We obtained ultrapure water (18.2 MΩ cm) from a Millipore Simplicity^®^ purification system (Darmstadt, Germany) for all extraction and optimization protocols. Regarding the synthesis materials, we employed MMT K10 (Sigma-Aldrich, product no. 281522) as our inorganic support, and acquired the fungal strain *P. ostreatus* 1005 from the Pennsylvania State University Mushroom Culture Collection (USA). Finally, the culture medium used to grow the fungus was prepared with 30 g L^−1^ glucose (C_6_H_12_O_6_; ACS Chemicals), 6 g L^−1^ yeast extract (Sigma-Aldrich), 0.5 g L^−1^ MgSO_4_ (Sigma-Aldrich), 0.5 g L^−1^ K_2_HPO_4_ (Sigma-Aldrich), and 0.1 g L^−1^ CaCl_2_ (Sigma-Aldrich), following the established formulation described by Montoya [32].

#### Hybrid material preparation and characterization

The biohybrid material was synthesized using a tailored in situ biological growth strategy, cultivating *P. ostreatus* in the direct presence of MMT. As the fungal culture developed, the expanding mycelial network progressively entrapped the clay microparticles, yielding a structurally integrated fungal–clay hybrid matrix. In a typical preparation, we dispersed 2.0 g of MMT into 200-mL Erlenmeyer flasks containing 50 mL of sterile culture medium. We then inoculated the flasks with ten 8-mm-diameter agar plugs harvested from the margins of actively growing mycelium. We incubated the cultures at 25 °C for 8 days, and subsequently agitated them using an Actum heavy duty HD-4000-CT orbital shaker at 101 rpm for another 8 days to promote strong interaction between the clay and the biomass. After this incubation period, we separated the extracellular medium from the hybrid biomass via filtration. After collecting the filtrate, we thoroughly washed the recovered biohybrid with ultrapure water to remove any remaining culture medium and loosely bound clay particles.

To examine the surface topography and morphology of the biohybrid, we performed scanning electron microscopy (SEM) using a Helios 5 PFIB CXe field-emission system (Thermo Fisher Scientific). Concurrently, we mapped the elemental distribution via energy-dispersive X-ray spectroscopy (EDS) with a Thermo Scientific Pathfinder Alpine system equipped with an UltraDry detector. We operated the microscope at an accelerating voltage of 30 kV, which provides a spatial resolution of 0.6 nm. Finally, we evaluated the textural properties of the material through nitrogen adsorption–desorption isotherms at 77 K using a Micromeritics ASAP 2020 Plus automatic adsorption analyzer, calculating the specific surface area via the Brunauer–Emmett–Teller (BET) method.

#### Instruments and software

We performed the chromatographic separations and mass spectrometric analyses using an Agilent 6890 N gas chromatograph coupled to an Agilent 5973 mass-selective detector (GC–MS). For the analytical separation, we used a DB-5ms capillary column (30 m × 0.25 mm i.d., 0.25 μm film thickness; Agilent Technologies, Santa Clara, CA, USA). We injected a 1-μL aliquot of each derivatized extract in split mode (split ratio 1:1) with the injector temperature set at 250 °C. Our oven temperature program was adapted from the method reported by Manzo et al. [33]. We started the program at 75 °C (held for 1 min), ramped the temperature to 250 °C at 25 °C min^−1^ (held for 1 min), and finally raised it to 300 °C at 10 °C min^−1^ with a final hold of 4 min. We maintained high-purity helium (99.999%) as the carrier gas at a constant flow rate of 1.0 mL min^−1^. Additionally, we set a solvent delay of 8 min, while keeping the transfer line and the electron ionization (EI, 70 eV) ion source temperatures strictly at 290 and 280 °C, respectively. For quantification, we operated the mass spectrometer in selected ion monitoring (SIM) mode. For the microextraction setups, we carried out all RDSE experiments using a custom Teflon extraction disk (1.5-cm diameter) housing a miniature Teflon-coated magnetic stir bar (VWR International, Radnor, PA, USA). We monitored and adjusted the pH of our solutions using an inoLab 7110 digital pH meter (WTW, Weilheim, Germany).

Finally, we performed the chemometric optimization through a Doehlert experimental design to thoroughly evaluate how the selected variables and their interactions affected extraction efficiency. We carried out the experimental design, statistical evaluation, and data processing within the R environment (R Foundation for Statistical Computing, Vienna, Austria, 2024), developing the specialized analysis scripts with coding assistance from Gemini 3.0 Flash [34].

#### RDSE extraction for the hybrid material

We performed a series of RDSE experiments to evaluate and compare the extraction efficiency of our synthesized biohybrid material against commercial PSA phases under identical conditions. For each run, we packed 50.0 mg of sorbent into the cavity of the Teflon extraction disk, which corresponds to its maximum capacity. Before starting the extraction, we activated the biohybrid material by rotating the disk in 5.0 mL of ultrapure water at 1100 rpm for 5 min. For comparison, we conditioned the commercial PSA sorbent following the precise protocol established by Honda et al. [35]. For the extraction step, we used 10.0-mL aqueous samples spiked with 5 µg L^−1^ of each steroid hormone, maintaining the disk rotational speed at 1100 rpm. We kept the operative conditions for the biohybrid material at their pre-optimized values to ensure a rigorous and fair performance comparison with the PSA reference phase. Once the extraction was complete, we desorbed the analytes by rotating the disk in 5 mL of high-purity methanol at 1100 rpm for 5 min. We then evaporated the resulting methanolic eluate to complete dryness under a controlled stream of nitrogen, and derivatized the dry residue using 100 µL of BSTFA at 80 °C for 30 min. Finally, we transferred the derivatized extracts into low-volume vial inserts for immediate GC–MS analysis. To guarantee statistical reproducibility, we executed all experimental sequences in triplicate (*n* = 3).

#### Optimization of the RDSE method

We evaluated the mass-transfer and partition dynamics within our RDSE system by systematically checking the primary chemical and hydrodynamic variables. Based on previous literature and our own preliminary screening tests, we initially identified disk rotational speed, sample volume, and extraction time as the most influential factors [22, 30]. However, our initial screening showed that most of these hydrodynamic factors had statistically nonsignificant effects within the tested ranges. For this reason, we decided to fix the sample volume at a constant value and focus our multivariate optimization strictly on two core variables: extraction contact time (X_1_) and desorption time (X_2_).

To achieve reliable and statistically sound optimization, we implemented a Doehlert experimental design matrix. This design encompassed nine randomized experimental runs (Table 1), which provided a balanced, uniform exploration of the experimental domain. One of the main practical advantages of this chemometric approach is that it allowed us to simultaneously estimate linear, cross-interaction, and second-order quadratic effects while significantly reducing the total number of laboratory experiments needed [36]. For our analytical response (Y), we chose the cumulative average of the chromatographic peak areas of the four target estrogens (E1, E2, E3, and EE2) obtained in each run. This multi-analyte strategy allowed us to holistically maximize the overall extraction yield rather than optimizing individual compound trajectories one by one. We rigorously evaluated the mathematical fit of the polynomial model using analysis of variance (ANOVA), considering *p*-values below 0.05 as statistically significant, and subsequently applied response surface methodology (RSM) to map the optimal conditions [37].

**Table 1:** Doehlert experimental design matrix with coded levels and corresponding actual values for the multivariate optimization of the RDSE–GC–MS method

Run	Coded values		Real values	
	X_1_	X_2_	Extraction time (min)	Desorption time (min)
1	0.5	0.866	75	50
2	−0.5	−0.866	45	10
3	0	0	60	20
4	−1	0	30	20
5	0	0	60	20
6	0.5	−0.866	75	10
7	1	0	90	20
8	0	0	60	20
9	−0.5	0.866	45	50

Regarding the wastewater samples, we observed highly stable native pH between 6.9 and 7.2, which remained virtually unaltered even after analyte fortification. Therefore, we performed all initial experiments at this native pH. Since these target steroid hormones have acid dissociation constants (pKa) between 10.2 and 10.8 [31], they exist predominantly in their non-ionized, neutral forms under these near-neutral conditions, which naturally favors hydrophobic interactions with our sorbent. To better understand the role of matrix pH on extraction thermodynamics, we later carried out comparative experiments using fortified water samples adjusted to pH 12.5, keeping our optimized extraction (75 min) and desorption (50 min) intervals constant.

#### Figures of merit and validation of the method in real samples

We validated the analytical performance of our optimized RDSE–GC–MS method following international quality guidelines. To determine the linear dynamic range, linearity, and sensitivity, we constructed matrix-matched calibration curves by spiking effluent wastewater samples with the target steroid hormones over a concentration range of 0–100 µg L^−1^ (specifically at 0, 2, 15, 30, 45, 60, 75 and 100 µg L^−1^). We analyzed each concentration level in triplicate (*n* = 3). We calculated the limits of detection (LOD) and quantification (LOQ) using a signal-to-noise ratio (S/N) approach, defining them as the analyte concentrations that yielded S/N ratios of 3 and 10, respectively, relative to the baseline noise of blank matrix extracts. We expressed the precision of the method as the relative standard deviation (%RSD, *n* = 3), while we evaluated accuracy through recovery percentages (%Re) [22].

We calculated the extraction recovery using Eq. 1:

$$Re\left(\%\right)=\left(\frac{\left(sample \;real+addition\right)-sample \;real}{addition}\right)\times 100.$$

For real-world environmental application, we collected raw influent wastewater samples from an HWW treatment system in Manizales, Colombia. We gathered the samples in amber glass bottles, immediately filtered them through a 0.45-µm polytetrafluoroethylene (PTFE) membrane to remove suspended solids, and stored them in the dark at 4 °C until analysis. We then packed our synthesized MMT–fungus biohybrid into the RDSE devices and operated them under the optimized chemometric conditions. Finally, we processed 10.0-mL aliquots of the filtered wastewater following the developed analytical protocol. To determine the concentration efficiency, we calculated the effective experimental enrichment factor (EF) using Eq. 2:

$$EF = \frac{{V}_{sample}}{{V}_{solvent\; extraction}}\times \frac{\%{R}_{e}}{100},$$

where *V*_*sample*_ is the initial volume of the aqueous sample (10 mL), *V*_*solvent extraction*_ represents the final volume of the reconstituted eluate before derivatization, and %Re corresponds to the experimental recovery achieved under our optimized conditions.

#### Sustainability, applicability, and innovation assessment of the MMT–fungus hybrid

To evaluate the environmental footprint of our RDSE–GC–MS method, we quantified its sustainability profile using the AGREEprep metric tool, strictly following the criteria established by Pena-Pereira et al. [38]. This multi-criteria assessment allowed us to systematically check solvent and reagent toxicity, hazardous waste generation, energy consumption, and our overall adherence to the core principles of green analytical chemistry. We scored each sequential step of the microextraction, conditioning, and elution protocols individually to get a clear, comprehensive picture of the method’s environmental performance. Beyond green chemistry, we evaluated the practical utility and operational feasibility of our method for routine laboratory work using the blue applicability grade index (BAGI) [39]. This index offers a standardized, objective framework for assessing key multidimensional parameters that matter in daily laboratory operations, including ease of use, cost-effectiveness, sample throughput, and instrumental accessibility. Finally, we appraised the degree of originality and technological advancement introduced by our analytical strategy using the violet innovation grade index (VIGI) [40]. This tool systematically evaluates the methodological uniqueness of our workflow, our strategic integration of the novel biohybrid material, and the overall technological progress this method contributes compared to conventional state-of-the-art setups.

## Results and discussion

### Characterization of the hybrid material

**Fig. 1:**
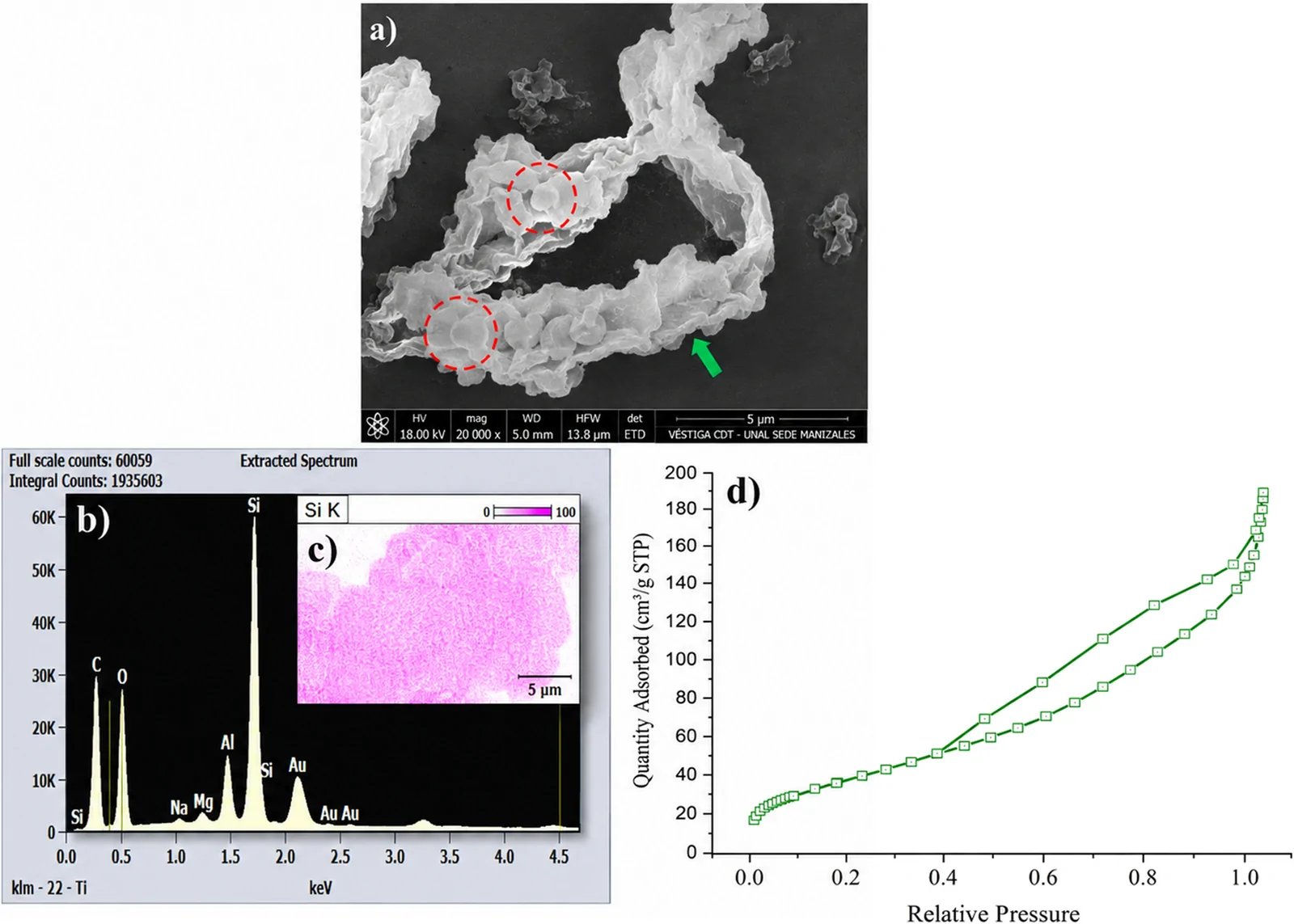
Characterization of the hybrid material: **a** SEM image, **b** EDS, **c** elemental mapping of Si; **d** BET sorptometry curve

To understand how the MMT and the fungus structured themselves into a single hybrid material, we analyzed its morphology and composition using SEM and EDS. Figure 1a clearly shows the hybrid’s architecture, where the typical flake-like layers of MMT [13, 23] are distributed along the dense network of fungal mycelia. Even with particles of different sizes, the material successfully retains the filamentous framework characteristic of the *P. ostreatus* mycelium, matching what has been reported for the fungus [41]. We attribute the formation of this hybrid mainly to natural electrostatic interactions. The net negative charge of the MMT sheets interacts spontaneously with the protonated amino and hydroxyl groups on the fungal cell wall. This affinity allows the clay platelets to self-assemble onto the hyphae, creating a stable, well-integrated, and highly robust network.

The EDS analysis of selected regions (Fig. 1b) confirmed the presence of C, O, Na, Mg, Al, and Si in the hybrid. The strongest signal came from the (EDS) Si Kα (~1.74 keV), confirming that the silicate phase from the MMT is the dominant component. The preservation of the Al signal shows that the clay’s framework does not degrade during fungal growth, while the minor Na and Mg signals correspond to the exchangeable interlayer cations typically found in natural MMT. As expected, the Au signal is a background artifact from the conductive coating needed for SEM imaging. Looking at the elemental mapping for Si (Fig. 1c), the homogeneous distribution across the scanned area (5-μm scale bar) proves that the clay remains well dispersed and non-aggregated within the biopolymer network even after colonization.

Regarding its textural properties, the N_2_ adsorption–desorption profiles (Fig. 1d) showed a typical IUPAC Type IV isotherm with a hysteresis loop at intermediate-to-high relative pressures, which is a standard indicator of mesoporous materials with slit-like pores [42]. After hybridization, the BET specific surface area decreased from 225.12 m^2^g^−1^ (for pristine MMT K10) to 148.06 m^2^g^−1^ in the hybrid. Since the fungal biomass alone showed a very low surface area (2.23 m^2^g^−1^), this reduction is logical and points to a partial blocking of the clay pores.

However, the hybrid still preserves a substantial portion of its original porosity. This is a critical feature for analytical applications because it ensures that the active adsorption sites remain highly accessible. Furthermore, this mesoporous framework acts as a clear advantage, as it facilitates unhindered analyte diffusion and significantly improves mass transfer during extraction. At the same time, the fungus introduces a high density of functional groups—such as amines, hydroxyls, and carboxyls—that can actively participate in hydrogen bonding. Moreover, the extracellular oxidative enzymes associated with the fungus may contribute to a partial transformation of the steroid structures [27–29, 43]. Ultimately, the hybrid material successfully combines structural advantages (surface area and mesoporosity) with rich chemical functionality (polar interaction sites and possible cation–π interactions) and potential biocatalytic activity. This combination fully supports its dual role as both an effective adsorbent and a reactive platform for transforming steroid hormones.

### Estrogen extraction using RDSE

We optimized the RDSE protocol using a multivariable Doehlert experimental design. Second-order polynomial quadratic models were used to correlate the extraction efficiency—measured as chromatographic peak areas—of the four target estrogens (E1, E2, EE2, and E3) against extraction time (X_1_) and desorption time (X_2_). The analysis of variance (ANOVA) confirmed that the quadratic frameworks are statistically significant for all analytes (Table S1), yielding precise *p*-values of 0.0087 for E1, < 0.0001 for E2, 0.0005 for EE2, and 0.0064 for E3. These values demonstrate that the models are highly reliable for mapping and predicting the analytical response within our experimental domain.

**Fig. 2:**
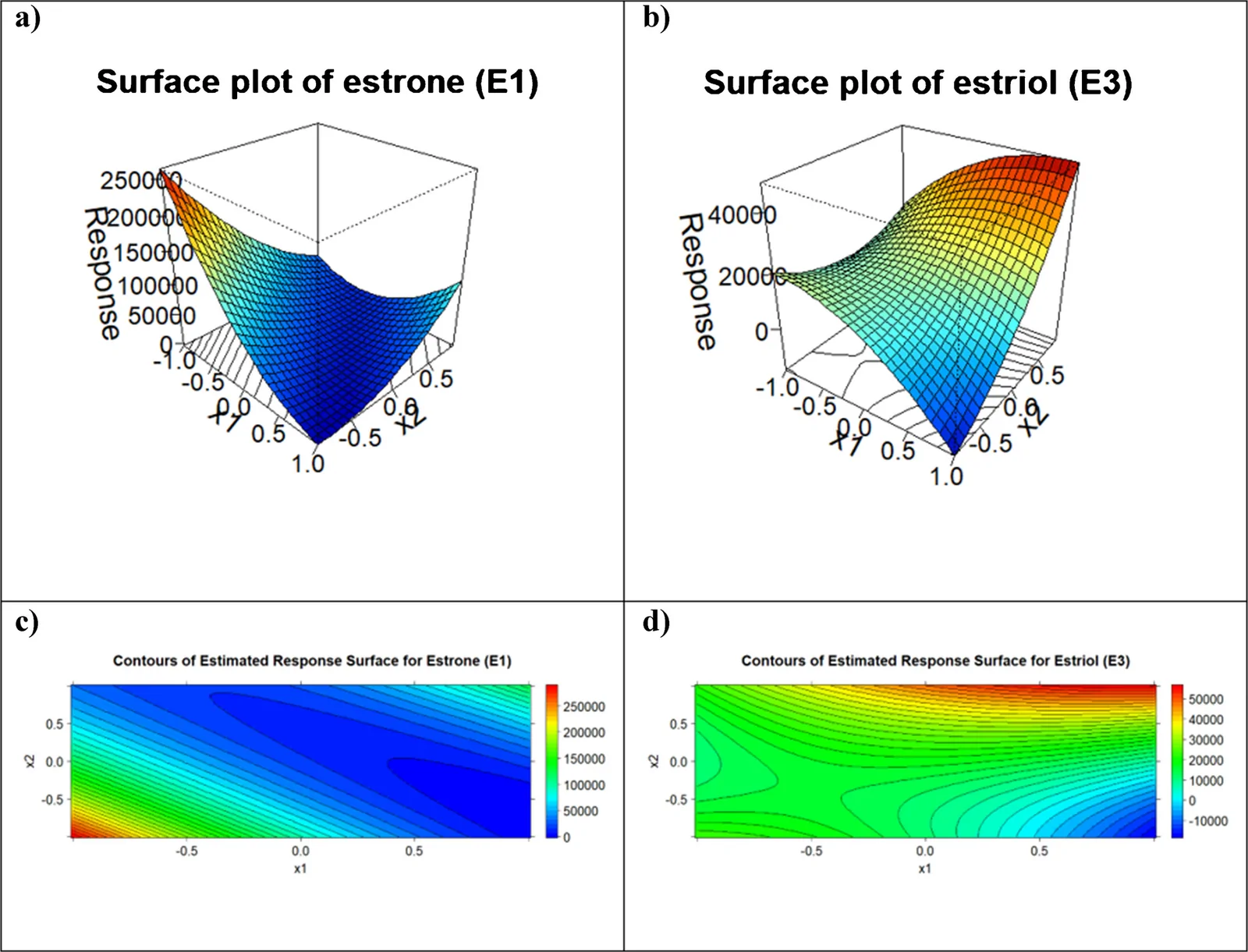
Response surface plots for **a** E1 and **b** E3, and corresponding contour plots for **c** E1 and **d** E3, as a function of extraction time (X_1_) and desorption time (X_2_) using coded factors, obtained from the Doehlert experimental design

The response surface plots and their corresponding contour maps (Fig. 2) clearly illustrate how both independent variables jointly influence the extraction yields of estrone (E1) and estriol (E3). Across the selected design space, E1 consistently showed higher extraction efficiency than E3. Notably, the pronounced curvature of the response surfaces, combined with the diagonally oriented, non-parallel contour lines (Fig. 2a–d), underscores a strong interaction effect X_1_ × X_2_. This clear interdependence proves that the extraction system cannot be properly optimized through simple univariate adjustments, fully justifying our use of second-order response surface methodology (RSM). In all cases, the models displayed coefficients of determination (*R*^2^) above 0.90 and adequate precision values greater than 4, confirming an excellent signal-to-noise ratio and strong predictive performance [44, 45]. To establish an ideal operational balance for simultaneous multi-analyte extraction, we applied a global desirability approach (D). Deconvolution of the desirability function identified the optimal coordinates at an extraction time of 75 min (coded X_1_ = 0.5) and a desorption time of 50 min (coded X_2_ ≈ 0.866), achieving a global desirability index of D = 0.936. The individual chromatographic peak areas for all experimental runs are presented in Table S5 (Supplementary Information). This optimized point represents a balanced compromise that successfully maximizes the concurrent recovery of all four hormones by leveraging the dual sorptive properties of the MMT–fungus hybrid phase.

We observed a clear relationship between the topographical profiles of the response surfaces and the structural polarities of the estrogens, which are described by their respective octanol–water partition coefficients (log *K*_*ow*_, Table S4). Among the analytes, E3 displayed unique mass-transfer behavior; as shown in Fig. 2b, it exhibits a convex response surface with its maximum located toward the central-to-high region of the experimental domain. Conversely, E1 (Fig. 2a) presents a concave profile with maxima shifted toward the boundaries of the design space—a trend closely mirrored by E2 and EE2. This behavioral divergence is consistent with the higher polarity and lower hydrophobicity of E3 (log *K*_*ow*_ ≈ 3.0–3.5) (X_1_) and desorption time (X_2_) than the more lipophilic estrogens. The observed curvature in the response surfaces and contours justifies the use of second-order quadratic models. A Significant interaction between X_1_ and X_2_ is evident from the non-parallel contour lines across all four hormones. Furthermore, the changing spacing of the contour lines directly reflects how rapidly the analytical response varies across the experimental coordinates.

These structural and kinetic correlations are further supported by the predicted optimal conditions calculated from the models (Table 2). While E1 and EE2 reached their maximum responses at identical coordinates (−0.5, −0.866)—corresponding to shorter operational times—E2 required longer extraction periods, reaching its optimum at the domain boundary (1.0, 0.000). In contrast, E3 optimization occurred at distinct coordinates (0.5, 0.866), highlighting that its recovery is more heavily restricted by desorption kinetics. The overall superior extraction efficiency recorded for E1 over E3 matches its higher lipophilicity (log *K*_*ow*_ ≈ 3.5–4.5), which promotes stronger hydrophobic interactions with both the carbonaceous fungal biomass and the siloxane surface of the MMT matrix.

**Table 2:** Predicted optimal conditions and maximum responses for each estrogen from the response surface models

Hormone	Maximum response	Coordinate (X_1_, X_2_)
E1	158,994.3	(−0.5, −0.866)
E2	745,110.0	(1.0, 0.000)
EE2	11,559.3	(−0.5, −0.866)
E3	48,956.0	(0.5, 0.866)

From a mechanistic standpoint, the response surfaces indicate that estrogen retention onto the MMT–fungus hybrid is driven by a cooperative network of non-covalent interactions. Hydrogen bonding and cation–π interactions associated with the inorganic montmorillonite layers act as primary retention vectors, while the integrated fungal biomass contributes complementary hydrophobic micro-domains and supplementary hydrogen-bonding donor/acceptor sites. Together, these features establish a synergistic analytical platform capable of selective steroid hormone retention based on molecular polarity [46].

### Application of the method in real samples and figures of merit

To test how the method performs in the real world, we validated our optimized RDSE–GC–MS protocol using HWW collected from a treatment plant in Manizales (Colombia). We chose this specific matrix because its chemical complexity and heavy organic background represent a significant challenge for trace analysis. We prepared matrix-matched calibration curves by spiking multi-standard hormone solutions directly into the wastewater samples across a range of 0–100 µg L^−1^. When we processed 10-mL aliquots under our optimized RDSE conditions, the analytical signal increased proportionally with concentration in all cases. This strong linear response confirms that the method is highly robust and perfectly capable of handling trace-level determination of these hormones even in heavily fouled environmental matrices.

To get a clearer picture of its performance, we ran a side-by-side comparison between our MMT–fungus hybrid sorbent and a conventional PSA phase under identical RDSE–GC–MS conditions. We evaluated standard benchmarking figures of merit, including limits of detection (LOD), limits of quantification (LOQ), relative recoveries (%R), coefficients of determination (*R*^2^), precision (as %RSD), and enrichment factors (EF). As summarized in Table 3, the MMT–fungus hybrid consistently outperformed the commercial PSA phase across almost all analytical parameters. The most remarkable improvement was in sensitivity, particularly for estriol (E3).

**Table 3:** Analytical figures of merit for the proposed MMT–fungus hybrid method compared with a commercial PSA sorbent in real wastewater

Analyte	MMT–fungus hybrid (GC–MS)							PSA			
								(GC–MS)			
	LOD	LOQ	%R (*n* = 3)	LDR (ng L^−1^)	Precision %RSD	*R*^2^	EF	LOD	LOQ	%R	EF
	(ng L^−1^)	(ng L^−1^)					%	(ng L^−1^)	(ng L^−1^)	(*n*= 5)	%
E1	6.92	20.0	99.99	0–100	2.9	0.9562	99.9	1.06	3.52	129.98	130.0
E2	9.08	20.0	94.69	0–100	8.5	0.9778	94.6	0.72	2.38	99.99	99.9
EE2	1.44	4.76	100	0–100	7.6	0.9947	100.0	2.63	8.70	85.96	85.1
E3	1.11	3.66	99.98	0–100	3.7	0.9958	99.9	3.22	104.24	48.9	48.9

LOD and LOQ values for the hybrid sorbent were calculated as 3 and 10 times the signal-to-noise ratio, respectively, from matrix-matched standards. Recoveries were determined at a spiking level within the linear range. *EF* enrichment factor

The analytical performance of the MMT–fungus hybrid sorbent was compared with that of a conventional PSA sorbent under identical RDSE–GC–MS conditions (Table 3). Comparison of the analytical parameters (Table 3) showed that the MMT–fungus biohybrid provided improved sensitivity for most target estrogens. For 17α-ethinylestradiol (EE2), LOD and LOQ improved from 2.63 and 8.7 ng L^−1^ (PSA) to 1.44 and 4.76 ng L^−1^ (hybrid). For the highly polar estriol (E3), the enhancement was even greater, with LOD/LOQ values of 1.11/3.66 ng L^−1^ versus 3.22/104.24 ng L^−1^ for PSA (~28-fold lower). Competitive LODs were obtained for estrone (E1, 6.92 ng L^−1^) and 17β-estradiol (E2, 9.08 ng L^−1^). Excellent linearity was observed in HWW (*R*^2^ = 0.9562–0.9947).

We observed a very similar trend for 17α-ethinylestradiol (EE2), where the detection limits were also substantially lower than those of the PSA control. At the same time, the hybrid maintained highly competitive limits for estrone (E1) and 17β-estradiol (E2), confirming that it performs effectively across a wide range of polarities. Matrix-matched linearity in the wastewater was excellent (*R*^2^ between 0.9562 and 0.9958), showing that the method remains reliable and accurate despite the heavy background interference. When we looked at extraction yields, the MMT–fungus hybrid gave near-quantitative and highly reproducible recoveries for nearly all analytes (≥ 99.9% for E1, EE2, and E3; 95% for E2; *n* = 3), clearly outperforming PSA, particularly for EE2 (100% vs. 86%) and E3 (99% vs. 49%). We attribute this enhanced performance to the synergistic chemistry of our hybrid material. While the MMT core provides a high surface area and structural stability, the *P. ostreatus* biomass introduces a rich variety of functional groups like hydroxyl, carboxyl, and amide moieties. Together, these components trigger multiple concurrent retention mechanisms—predominantly hydrogen bonding and cation–π interactions—that are far more effective for capturing steroid rings than the basic anion-exchange interactions offered by PSA. As a result, we achieve much cleaner extracts and better signal-to-noise ratios at trace levels. Regarding preconcentration, the enrichment factors (EF) obtained with the hybrid material ranged from 100.0 to 105.7, which are comparable to or slightly higher than those of the PSA phase (94.6–100.0). This indicates that while both sorbents enrich the analytes effectively under rotating-disk dynamics, our hybrid system delivers far more consistent, selective, and reproducible performance across all target hormones. Ultimately, these results establish the MMT–fungus hybrid as a more balanced, chemically robust, and sustainable alternative to commercial sorbents, making it an excellent candidate for the environmental monitoring of endocrine-disrupting compounds.

### Comparative evaluation of sorbent performance and matrix effects

**Fig. 3:**
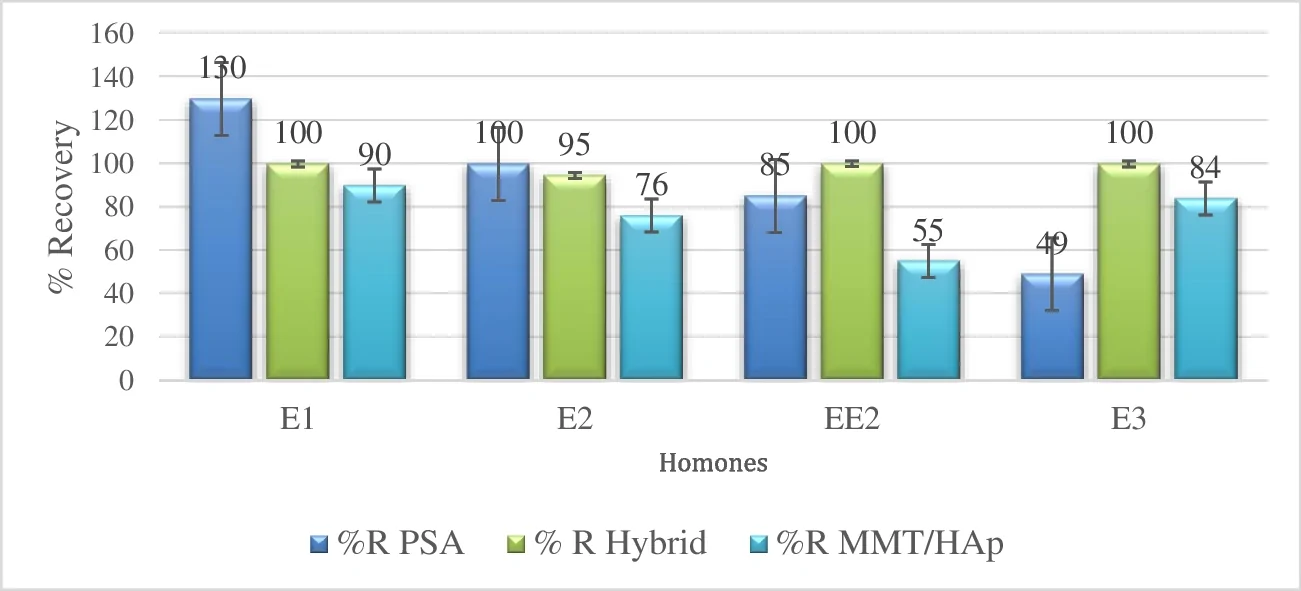
Variation in recovery percentages of estrogenic hormones according to adsorbent type using RDSE for MMT–fungus hybrid, PSA, and MMT/HAp

To benchmark the extraction capability of the novel MMT–fungus hybrid, we compared its relative recovery profiles against conventional PSA and hydroxyapatite-modified montmorillonite (MMT/HAp) sorbents under identical RDSE conditions (Fig. 3). The MMT–fungus hybrid demonstrated remarkably consistent and robust extraction performance, yielding quantitative recoveries between 95% and 100% for all target analytes. This uniform behavior indicates that the mass-transfer and retention mechanisms achieved by the hybrid phase are largely independent of the individual physicochemical differences and polarities of the studied estrogens.

In stark contrast, the commercial PSA phase displayed a highly irregular and variable extraction profile. While PSA achieved acceptable recoveries for 17β-estradiol (99%) and 17α-ethinylestradiol (86%), its recovery for estriol dropped significantly to 49%, and estrone conversely exhibited an anomalous value of 130%. This over-recovery exceeding 100% for estrone points to a severe positive matrix effect, typically arising from co-eluting matrix interferences that induce signal enhancement during gas chromatographic vaporization or ionization. This trend is consistent with previous reports utilizing complex HWW matrices, where matrix effects are known to fluctuate widely from −67% to +230% [14]. Consequently, these deviations underscore the fact that raw recovery values in heavily fouled environmental samples cannot be interpreted solely as indicators of net extraction efficiency; rather, they result from the combined influence of sorbent thermodynamic affinity, matrix-induced signal modulation, and overall methodological uncertainty. To bypass these systematic biases and guarantee quantitative accuracy, we found the implementation of a standard addition calibration protocol to be essential [14].

From a mechanistic perspective, the operational superiority of the hybrid material over both PSA and MMT/HAp can be explained by looking at their respective surface chemistries. Adsorption onto the MMT component is driven predominantly by its highly polar surface framework [47], and the retention of steroid hormones is mediated by localized hydrogen bonding between the steroidal hydroxyl and carbonyl functional groups and the clay’s surface silanol (Si–OH) and aluminol (Al–OH) sites, complemented by structural stabilization via dipole–dipole interactions. For these specific aromatic analytes, cation–π interactions established between the electron-rich phenolic A-ring of the estrogens and the exchangeable interlayer Ca^2+^ ions further lock the molecules within the clay galleries [14, 48]. While the basic anion-exchange pathways of the PSA sorbent lack the required selectivity to capture analytes across such a wide polarity range, the MMT/HAp sorbent exhibited only intermediate performance [14]. Specifically, MMT/HAp provided acceptable recoveries for estrone (90%) and estriol (84%) and a moderate yield for 17β-estradiol (76%), but a notably lower recovery for 17α-ethinylestradiol (55%). This specific reduction suggests a lower thermodynamic affinity toward the ethinyl moiety, which we attribute to steric hindrance that limits the accessibility of the active sites on the hydroxyapatite-modified MMT surface, thereby disrupting the stabilizing cation–π networks.

**Fig. 4:**
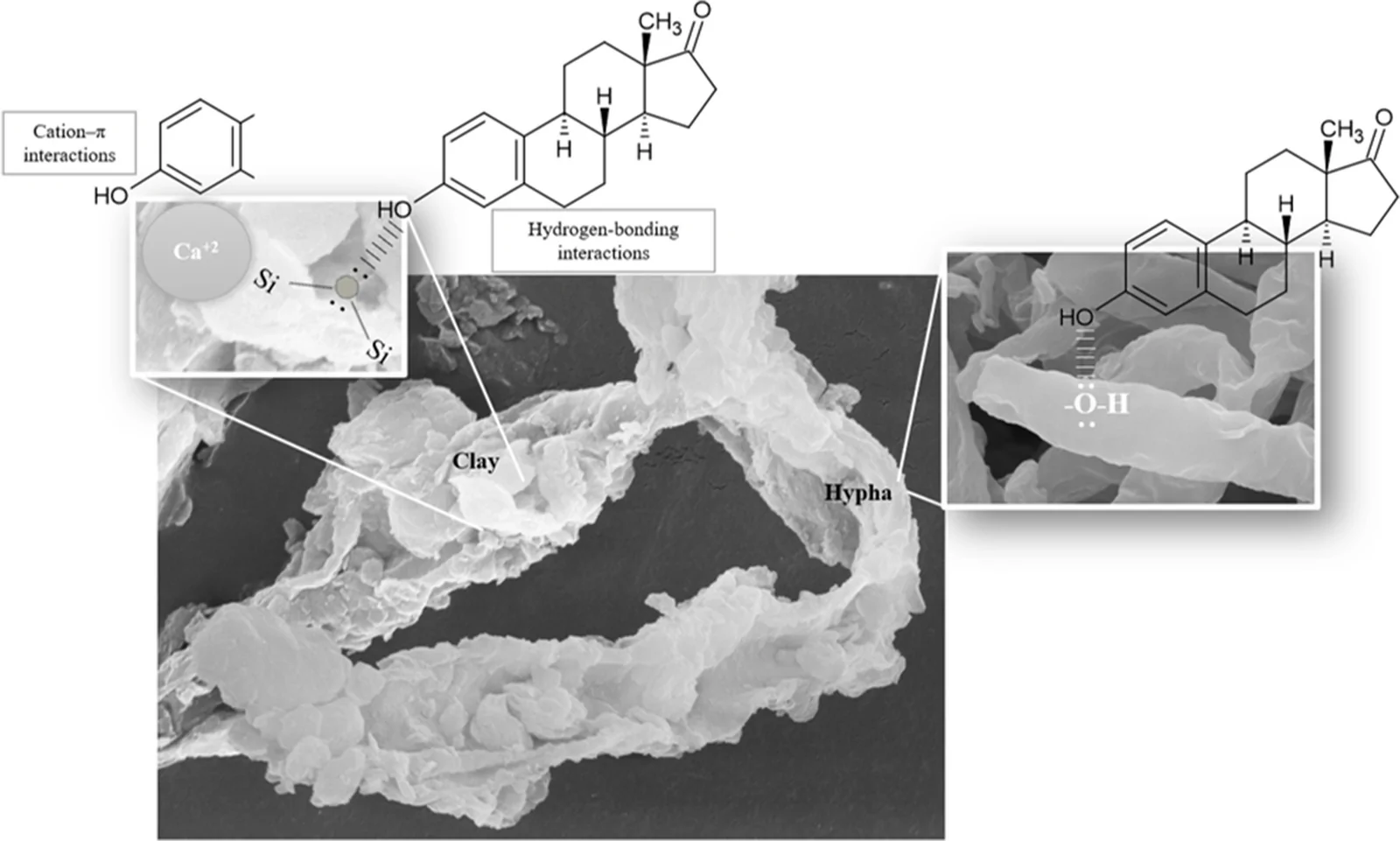
Schematic illustration of the adsorption mechanisms of estriol on the montmorillonite–fungal hyphae biohybrid material

Conversely, the integrated fungal biomass in our hybrid material successfully introduces a high density of supplementary carboxyl, hydroxyl, and amino functional groups. These biopolymers amplify the overall hydrogen-bonding network and increase the affinity of the sorptive phase without causing pore blockage or restricting access to the clay active centers [46]. The idealized adsorption mechanism for estriol onto the hybrid surface (Fig. 4) visually maps this cooperative matrix effect. Ultimately, the preservation of the native clay mesoporosity, high specific surface area, and homogeneous distribution of the aluminosilicate phase within the mycelial network work in synergy, ensuring efficient analyte transport, excellent mass-transfer kinetics, and reproducible chromatographic performance.

To place our method within the landscape of contemporary sample preparation strategies, we benchmarked its performance against representative methodologies reported for determining steroid estrogens in environmental waters (Table 4). This comparison covers a diverse array of sorbent-based configurations, including solid-phase extraction (SPE), online SPE, solid-phase microextraction (SPME), in-tube SPME, stir-bar sorptive extraction (SBSE), bar absorptive microextraction (BAμE), fabric-phase sorptive extraction (FPSE), continuous SPE, and alternative RDSE setups [13–21, 30, 49–51]. As expected, several protocols coupled with liquid chromatography–tandem mass spectrometry (LC–MS/MS) achieve lower detection thresholds, which is inherently due to the higher sensitivity of tandem mass spectrometers. Nevertheless, our RDSE–GC–MS method delivers highly competitive limits of detection between 1.1 and 9.1 ng L^−1^ and relative recoveries consistently spanning 95% to 100%. These values demonstrate that the developed protocol compares quite favorably with established sorbent-based extraction methods in the literature, offering an excellent analytical balance without strictly requiring ultra-trace tandem mass detection.

**Table 4:** Comparison of representative analytical methodologies reported for the determination of steroid estrogens in environmental waters and the proposed RDSE–GC–MS method based on the MMT–fungus biohybrid sorbent

Extraction technique	Sorbent/extraction phase	Detection system	LOD (ng L^−1^)	Recovery (%)	Sustainability features	Reference (DOI)
**Immunoaffinity extraction**	Antibody-based sorbent	LC/ESI–MS	0.07–0.18	>90	High selectivity; expensive sorbent preparation and limited reusability	[20]
**SPE**	tC18 + NH_2_ cartridges	LC–MS/MS	0.1–0.2	83–105	High sensitivity; several preparation steps and relatively high solvent consumption	[15]
**Online SPE**	Oasis HLB (hydrophilic–lipophilic balance)	LC–ESI–MS/MS	0.02–1.02	>74	Automated operation, excellent sensitivity; disposable cartridges	[16]
**SPE**	C18 cartridge	LC–MS/MS	2.5–19.2	80–130	Robust methodology; disposable cartridges and solvent consumption	[17]
**In-tube SPME**	Supel-Q porous layer open tubular (PLOT) capillary column	LC–MS/MS	2.7–11.7	86–103	Solvent-free, automated, and reusable extraction device	[19]
**SPME**	Polyacrylate (PA) fiber	GC–MS/MS	0.3–5	80–110	Solvent-free extraction; reusable fiber	[18]
**SBSE**	Polydimethylsiloxane (PDMS)-coated stir bar	TD–GC–MS	0.1–0.5	>90	Very high sensitivity; reusable sorptive phase	[21]
**BAµE**	Activated carbon	HPLC–DAD	50–100	93–101	Reusable extraction device; low solvent consumption	[49]
**FPSE**	Sol–gel-coated fabric	HPLC–FLD	20–42	89–98	Reusable fabric support; minimal solvent consumption	[50]
**RDSE**	Laminar cork	GC–MS	6–63	>63	Reduced solvent consumption but chlorinated solvent required	[30]
**Continuous SPE**	Oasis HLB	GC–MS	0.01–0.3	92–103	Good compromise between sensitivity and solvent consumption	[51]
**RDSE**	Ionic liquid-modified clay	HPLC–PDA	10–70	20–61	Low solvent consumption; ionic-liquid modification required	[13]
**RDSE**	Montmorillonite/hydroxyapatite	GC–MS	60–400	55–90	Natural sorbent, low solvent consumption; matrix effects reported	[14]
**RDSE (present work)**	**MMT–fungus biohybrid**	**GC–MS (SIM)**	**1.1–9.1**	**95–100**	**Natural biohybrid sorbent, reusable, low solvent consumption, AGREEpre*p* = 0.75, BAGI = 52.5, VIGI = 80**	Present work

*HPLC–DAD* high-performance liquid chromatography with diode-array detector, *HPLC–FLD* high-performance liquid chromatography with fluorescence detector, *LC/ESI–MS* liquid chromatography–electrospray ionization–mass spectrometry, *LC–ESI–MS/MS* liquid chromatography–electrospray ionization–tandem mass spectrometry, *TD–GC–MS* thermal desorption–gas chromatography–mass spectrometry

Beyond conventional figures of merit, this work introduces the MMT–fungus biohybrid as a novel sorptive phase for RDSE, expanding the range of sustainable and renewable materials available for disk-geometry microextraction techniques. We further evaluated its holistic profile using white analytical chemistry (WAC) metrics. The computed scores for AGREEprep (0.75), BAGI (52.5), and VIGI (80) point to a highly favorable balance between analytical performance and environmental sustainability, supporting the practical applicability of the method for routine estrogen monitoring in complex aqueous matrices. Overall, these findings clearly show that the composition of the sorptive phase plays a central role in governing RDSE performance. Among the materials evaluated, our MMT–fungus biohybrid provided the most consistent extraction behavior, yielding highly quantitative recoveries for all target estrogens regardless of their structural polarities. These results highlight the potential of this previously unreported biohybrid sorbent as a reliable, high-performance, and sustainable alternative for the extraction and determination of steroid estrogens in complex environmental waters.

To get a clear picture of the forces driving the extraction process, we evaluated the sorptive behavior of the target estrogens onto the MMT–fungus biohybrid from a molecular perspective. The real HWW samples had native pH values ranging tightly between 6.9 and 7.2. Under these near-neutral conditions, the target hormones—estrone (E1), 17β-estradiol (E2), 17α-ethinylestradiol (EE2), and estriol (E3)—exist almost entirely as uncharged, neutral molecules. This behavior is dictated by the acid dissociation constants (pKa) of their phenolic hydroxyl groups, which lie around 10.33–10.54 [52]. Additionally, the aliphatic hydroxyl groups on E3 have pKa values well above 14 [53], meaning they stay non-ionized across the investigated pH range. As a result, while the target steroid estrogens are essentially neutral at pH 7.0, raising the pH to 12.5 completely deprotonates the phenolic hydroxyl groups, shifting the analytes into negatively charged, highly hydrophilic phenolate species.

**Fig. 5:**
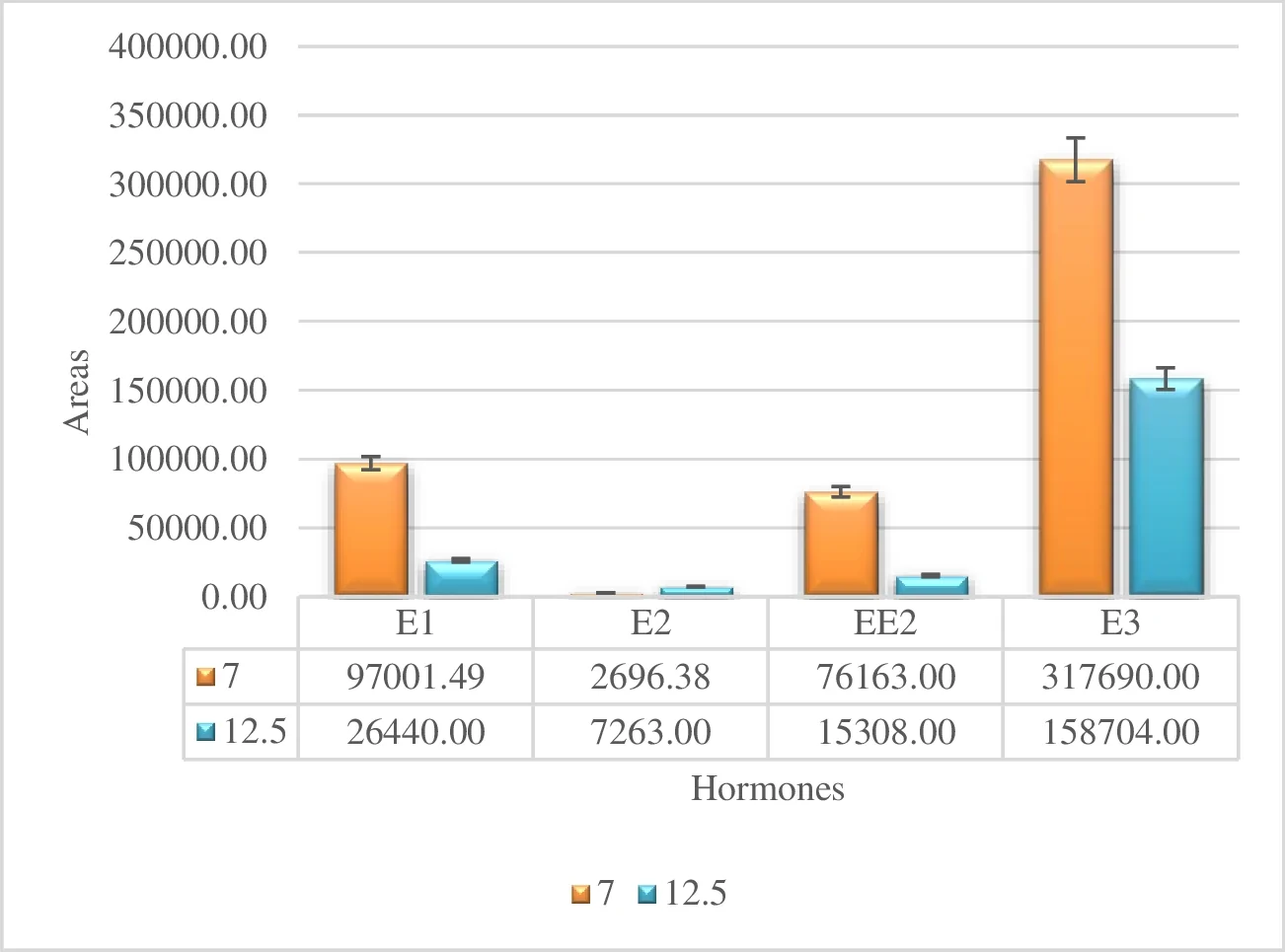
Comparison of extraction performance at pH 7.0 and 12.5 based on peak areas of estrogenic compounds

The experimental results shown in Fig. 5 reveal a dramatic drop in chromatographic peak areas when moving from a near-neutral pH to 12.5. This clear change confirms that hydrogen bonding acts as a primary driving force behind the extraction. When the estrogens turn into their negatively charged forms at pH 12.5, the electrostatic repulsion between the analytes and the net negative surface charge of both the clay sheets and the fungal biopolymers heavily suppresses retention. Interestingly, at pH 7.0, E2 showed slightly lower extraction yields than the other estrogens, a trend that correlates perfectly with its lower density of hydrogen-bond donor and acceptor sites. Within the MMT phase, the main groups involved in these interactions are the silanol (Si–OH) and aluminol (Al–OH) sites located at the particle edges. With pKa values between 7.0 and 8.5, these amphoteric edge groups can act as either hydrogen-bond donors or acceptors at a near-neutral pH. At the same time, the framework siloxane oxygen atoms (Si–O–Si) on the basal planes serve as permanent hydrogen-bond acceptors, creating an ideal surface network for anchoring the neutral steroid molecules [47].

The enhanced extraction performance of our MMT–fungus hybrid clearly comes from the excellent structural and chemical synergy between the clay mineral and the fungal biomass. The MMT core provides a high specific surface area, structured mesoporosity, and exchangeable Ca^2+^ interlayer ions that can establish stabilizing cation–π interactions with the aromatic A-ring of the steroids. Complementing this inorganic frame, the chitin- and glucan-rich fungal cell wall introduces massive abundance of primary/secondary amino and hydroxyl functional groups, expanding both the total number and the diversity of the active adsorption sites.

The structural hydroxyl groups at the C3 and C6 positions of the chitin chain have pKa values above 12, ensuring they remain protonated and neutral at the working wastewater pH. Meanwhile, the surface-accessible amino groups (pKa ≈ 6.2–6.5), which largely control the surface charge of the biomass, exist predominantly in their neutral deprotonated form –NH_2_ at pH 6.9–7.2 [54]. Under these specific conditions, pure electrostatic or coulombic interactions between the sorbent surface and the neutral estrogens are minimal. This forces the adsorption mechanism to be driven cooperatively by hydrogen-bonding networks, dipole–dipole stabilization, and hydrophobic partitioning into the cell-wall micro-domains. The convergence of these complementary, non-covalent pathways provides a logical and complete explanation for the outstanding extraction efficiency displayed by our MMT–fungus biohybrid material.

### Real sample analysis with hospital wastewater

**Fig. 6:**
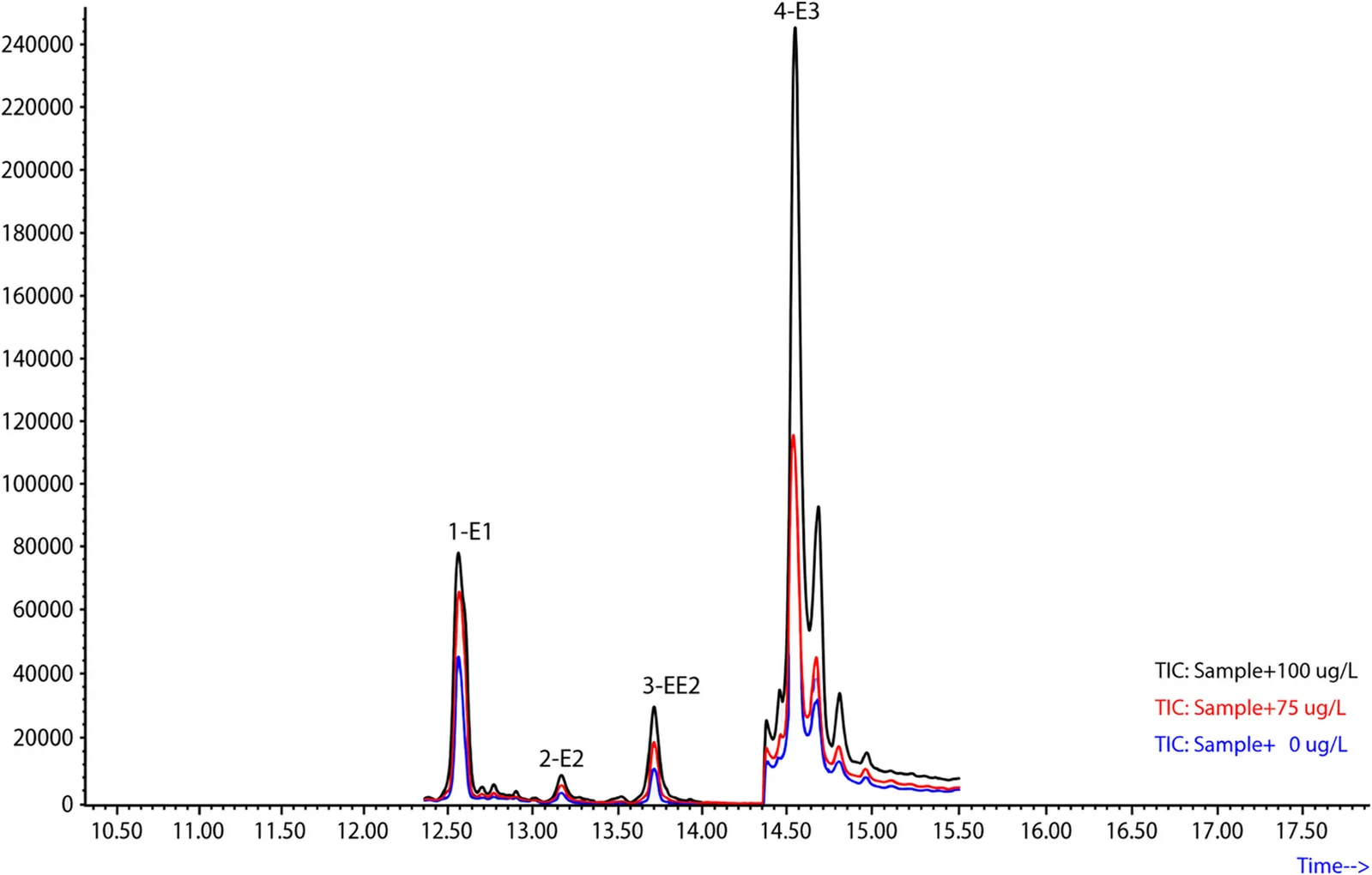
Representative total ion chromatograms (TIC) of WWTP extracts obtained with the MMT–fungus hybrid sorbent. Overlaid chromatograms correspond to unspiked sample (blue line) and samples spiked at 75 µg L^−1^ (red line) and 100 µg L^−1^ (black line). Peaks: 1 = E1 (estrone), 2 = E2 (17β-estradiol), 3 = EE2 (17α-ethinylestradiol), 4 = E3 (estriol).

The environmental applicability and performance of the optimized RDSE method were evaluated through the simultaneous determination of estrone (E1), 17β-estradiol (E2), 17α-ethinylestradiol (EE2), and estriol (E3) in raw HWW utilizing GC–MS. Figure 6 displays a representative total ion chromatogram (TIC) showcasing the chromatographic separation profiles. All four target compounds achieved satisfactory baseline resolution, with retention times recorded at 12.5 min (E1), 12.9 min (E2), 13.5 min (EE2), and 14.3 min (E3).

The overlay of the unspiked wastewater matrix with the matrix samples fortified at 75 and 100 µg L^−1^ demonstrated highly symmetrical peak shapes free of discernible co-eluting interferences. This clean background confirms that the multimodal sorptive mechanism of the MMT–fungus biohybrid provides an exceptionally selective clean-up phase, efficiently isolating trace-level steroids while discarding highly complex organic fractions from the wastewater background. Table 5 compiles the operational mass-to-charge ratios (*m*/*z*), exact retention windows, quantitative concentration levels, and precision data.

**Table 5:** Retention times, ions monitored, concentrations found in WWTP samples, Horwitz-predicted RSD, and experimental %RSD obtained with the MMT–fungus hybrid

Hormone	Retention time (min)	Quantification ion, qualification ion (m/z)	WWTP amount (ng L^−1^) MMT–fungus hybrid	Approximate Horwitz-predicted RSD (%) (*n*=3)	Experimental %RSD (MMT–fungus (*n*=3)
E1	12.5	257, 342	628	24.6	2.9
E2	12.9	285, 416	596	24.3	8.5
EE2	13.5	285, 368	195	28.7	7.6
E3	14.3	147, 345	853	22.9	3.7

All four target endocrine-disrupting steroids were positively identified and quantified in the authentic effluents, yielding concentration values of 628 ng L^−1^ (E1), 596 ng L^−1^ (E2), 195 ng L^−1^ (EE2), and 853 ng L^−1^ (E3). This concentration distribution accurately reflects typical occurrence profiles reported globally for natural and synthetic estrogens in urban aqueous environments receiving untreated clinical discharge.

Method precision was rigorously tested via intra-laboratory repeatability assays and expressed as the relative standard deviation %RSD (%RDS, *n* = 3). The calculated experimental precision values were remarkably low, yielding 2.9% for E1, 8.5% for E2, 7.6% for EE2, and 3.7% for E3.

To provide an objective statistical validation of the method’s precision, the experimental repeatability was evaluated against the predicted relative standard deviation (PRSD) calculated from the modified Horwitz equation: PRSD ≈ 2*C*^−0.15^, where *C* represents the mass fraction of the analyte. Within the ultra-trace concentration boundaries monitored in this work (approximately 2 × 10^−8^ to 8.5 × 10^−8^ g g^−1^), the experimental precision remained well within the theoretically acceptable Horwitz threshold, yielding narrow HorRat ratios between 0.12 and 0.35. Consequently, the resulting Horwitz ratio (HorRat) indices ranged between 0.12 and 0.35. Since these values sit substantially below the standard benchmark of unity (HorRat ≤ 1), the protocol demonstrates a level of repeatability that significantly surpasses conventional expectations for ultra-trace environmental assays. Furthermore, the complete chromatographic run was achieved in under 15 min, providing a rapid, high-throughput duty cycle without compromising the diagnostic reliability of the target and qualifier ions.

In summary, the developed analytical workflow demonstrates excellent robust performance when challenged by a heavily fouled HWW matrix. The low HorRat indices and narrow RSD distributions confirm the high repeatability of all coupled experimental stages, including microextraction, chemical derivatization, and GC–MS analysis. Despite the high loading of dissolved organic and inorganic species inherent to clinical effluents, no chemical matrix effect or significant peak distortion was observed under the optimized configurations. The combination of rapid separation kinetics, high precision thresholds, and excellent clean-up efficiency establishes the MMT–fungus biohybrid as a powerful, sustainable, and reliable solid-phase alternative for tracking steroidal micropollutants in complex environmental waters.

### Highly innovative MMT–fungus hybrid sorbent for precise trace determination of estrogenic steroids in wastewater: superior precision and green metrics

**Fig. 7:**
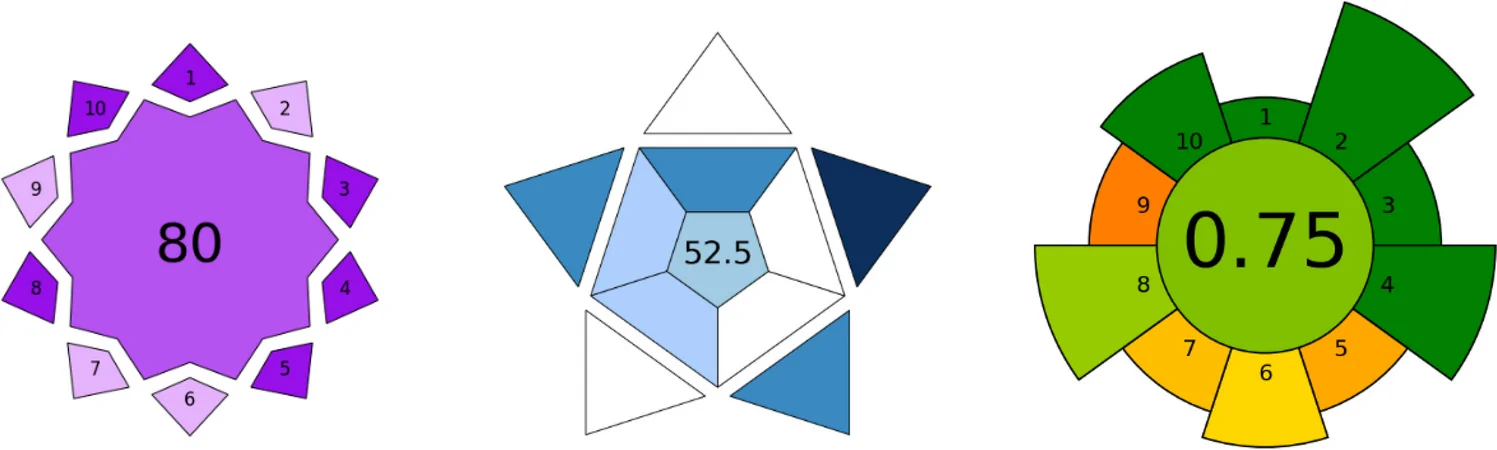
Combined evaluation of innovation (violet innovation grade index, VIGI), applicability (blue applicability grade index, BAGI) and AGREEprep of the MMT–fungus hybrid sorbent method

To evaluate the true environmental impact, operational practicality, and innovative nature of our RDSE method, we moved beyond classic figures of merit and tested the protocol against the AGREEprep, BAGI, and VIGI metrics. This multi-metric approach allows us to look at sample preparation from a holistic, modern perspective rather than relying on a single green score. As shown in Fig. 7, the developed method achieved an outstanding AGREEprep score of 0.75, demonstrating high compliance with the core principles of green analytical chemistry (GAC) (S2). This favorable profile is directly due to three key choices in our operational design: we significantly minimized organic solvent consumption during desorption, kept hazardous waste generation to a minimum, and utilized a completely renewable, bio-derived hybrid sorbent—all of which represent heavily weighted parameters within the AGREEprep evaluation framework [38].

To place these environmental metrics in a broader context, we compared our AGREEprep score against conventional microextraction techniques reported in the literature. For instance, configurations like microextraction by packed sorbent (MEPS) and solid-phase microextraction (SPME) typically exhibit lower sustainability scores of 0.55 and 0.53, respectively (Table S3, Supplementary Information) [38]. Furthermore, a wider survey of automated and manual microextraction methods reported values spanning a lower range from 0.22 to 0.65 (Table S3, Supplementary Information) [55]. While these literature benchmarks focus on diverse analytical applications rather than estrogen monitoring, they establish a clear baseline showing that our RDSE setup significantly improves the green nature of routine microextraction workflows.

We also benchmarked our indices against a recently reported RDSE method that uses a semi-synthetic MMT/HAp sorbent to extract estrogens from the same wastewater matrix (BAGI = 50; VIGI = 60) (Table S3, Supplementary Information) [14]. Our current protocol demonstrated superior metrics, achieving a BAGI value of 52.5 and a VIGI score of 80. These increments reflect better practical applicability and higher methodological innovation, which we attribute primarily to the transition from semi-synthetic phases to a completely bio-derived hybrid matrix. However, these variations should be interpreted with caution; both the BAGI and VIGI frameworks integrate multi-criteria algorithms that weigh device simplicity, operational steps, and innovation differently depending on the specific application.

When looking at the state-of-the-art methodologies for determining steroid estrogens in environmental waters, we noticed a clear trend. While most protocols provide adequate sensitivity and recoveries, they still rely heavily on commercial silica-bonded SPE cartridges, immunoaffinity phases, or sophisticated synthetic sorbents. As summarized in Table 4, these traditional workflows typically involve tedious multistep sample preparation sequences and require high volumes of toxic conditioning and elution solvents. Furthermore, comprehensive multi-metric evaluations (such as AGREEprep, BAGI, and VIGI) remain surprisingly scarce in current estrogen literature, which makes a systematic, side-by-side comparison of environmental impacts across published works quite difficult.

By incorporating a structured sustainability assessment alongside classical performance data, this study bridges the gap between analytical efficiency and green chemistry. The developed MMT–fungus RDSE strategy successfully couples an eco-friendly, bio-derived sorbent with a highly simplified, high-throughput extraction procedure without sacrificing the sensitivity required for trace-level hormone determination. Ultimately, the integration of these parameters establishes our method as a robust, legally viable, and sustainable alternative for tracking endocrine-disrupting steroids in heavily fouled wastewater, aligning routine environmental monitoring with modern green chemistry trends.

## Conclusions

In this study, a montmorillonite–*P. ostreatus* (MMT–fungus) biohybrid material was successfully developed and evaluated as an advanced sorptive phase for the rotating-disk sorptive extraction (RDSE) and simultaneous determination of four target steroid estrogens (E1, E2, EE2, and E3) in hospital wastewater via GC–MS. Distinct from prior investigations—including earlier studies utilizing alternative extraction phases—the primary novelty of this work resides in the synthesis, physicochemical characterization, and analytical validation of this specific biohybrid matrix, rather than in architectural modifications to the underlying RDSE methodology.

Structural and textural characterization confirmed that the hybrid matrix effectively preserves both the characteristic mesoporosity and high specific surface area original clay framework, while seamlessly integrating the dense, functionalized biopolymer network of the fungal hyphae. Mechanistic deconvolution revealed that estrogen retention onto this biohybrid phase is governed by a robust, cooperative network of non-covalent interactions. Under near-neutral operational pH conditions, where the target hormones exist in their neutral molecular forms, mass transfer and sorptive capture are primarily driven by localized hydrogen-bonding networks with both clay edge sites (Si–OH, Al–OH) and cell-wall functionalities (NH_2_, –OH), supplemented by stabilizing cation–π interactions involving exchangeable interlayer Ca^2+^ ions and the aromatic A-ring of the steroids.

Method validation demostrated excellent analytical performance: experimental precision (%RSD = 2.9–8.5%) sat substantially below the corresponding Horwitz-predicted values, yielding narrow HorRat distributions between 0.12 and 0.35, which mathematically confirms the robustness of the proposed analytical procedure. Furthermore, the application of this method to hospital wastewater treatment plant effluents confirmed the occurrence of natural and synthetic steroid estrogens at concentrations ranging from 195 to 853 ng L^−1^, demonstrating the practical reliability and applicability of the sorbent when challenged by heavily fouled environmental samples.

Beyond its competitive analytical figures of merit, the MMT–fungus biohybrid represents a highly sustainable alternative to conventional commercial sorbents. The material benefits from a straightforward biological synthesis, utilizes renewable raw materials, carries a low production cost, and exhibits a favorable environmental profile validated by white analytical chemistry (WAC) metrics (VIGI = 80, AGREEprep = 0.75, and BAGI = 52.5). In conclusion, this study establishes the MMT–fungus biohybrid as a powerful extraction material for analytical microextraction frameworks and provides a solid operational foundation for the future architectural development of sustainable biohybrid sorbents in environmental analysis.

## Supplementary Information

Below is the link to the electronic supplementary material.

Supplementary file1 (PDF 562 KB)

## Data Availability

Data will be made available on request.
