# Supplementary material for: Montmorillonite–fungal biomass hybrid as sustainable sorbent in rotating-disk sorptive extraction coupled to GC–MS for trace-level monitoring of estrogens in hospital wastewater
Source: Anal Bioanal Chem. 2026 Aug 26;418(21):7197–214. doi: 10.1007/s00216-026-06715-8 (PMC13658567; doi:10.1007/s00216-026-06715-8)
Supplement: Supplementary file 1 [file 216_2026_6715_MOESM1_ESM.pdf]

Suplementary 1

Table 1. ANOVA results and statistical validation parameters for estrone (E1).

Source	df	Sum of Squares	Mean Square	*F-value*	*p-value*	Significant
Model	5	2.215E+10	4.431E+9	31.05	0.0087	**
X_1-contact time (min)	1	7.419E+9	7.419E+9	51.99	0.0055	**
X_2-desorption time (min)	1	3.454E+9	3.454E+9	24.20	0.0161	*
X_1X_2	1	6.788E+9	6.788E+9	47.57	0.0062	**
X_1^2	1	3.262E+9	3.262E+9	16.78	0.0263	*
X_2^2	1	2.097E+9	2.097E+9	14.70	0.0313	*
Residual	3	4.281E+8	1.427E+8			
Lack of Fit	1	9.943E+6	9.943E+6	0.0476	0.8476	Not significant
Pure Error	2	4.181E+8	2.091E+8			

R^2 = 0.9810, Adj-R^2 = 0.9494, Pred-R^2 = 0.9425, CV = 14.02%, Adeq-Precision = 22.3067.

* $p < 0.05$
** $p < 0.01$
*** $p < 0.001$

Table 2. ANOVA results and statistical validation parameters for 17β-estradiol (E2).

Source	df	Sum of Squares	Mean Square	*F-value*	*p-value*	Significant
**Model**	5	8.220E+11	1.644E+11	2473.82	< 0.0001	**
X_1-contact time (min)	1	3.629E+11	3.629E+11	5460.72	< 0.0001	***
X_2-desorption time (min)	1	1.177E+11	1.177E+11	1770.55	< 0.0001	***
X_1X_2	1	1.586E+11	1.586E+11	2386.26	< 0.0001	***
X_1^2	1	1.787E+11	1.787E+11	2423.89	< 0.0001	***
X_2^2	1	2.177E+10	2.177E+10	327.67	0.0004	***
Residual	3	1.994E+8	6.645E+7			
Lack of Fit	1	1.631E+8	1.631E+8	9.0109	0.0954	Not significant
Pure Error	2	3.621E+7	1.811E+7			

R^2 = 0.9998, Adj-R^2 = 0.9994, Pred-R^2 = 0.9928, CV = 2.62%, Adeq-Precision = 157.9405
* $p < 0.05$
** $p < 0.01$
*** $p < 0.001$

Table 3. ANOVA results and statistical validation parameters for 17α-ethinylestradiol (EE2).

Source	df	Sum of Squares	Mean Square	*F-value*	*p-value*	Significant

**Model**	5	9.409E+7	1.882E+7	222.07	0.0005	
X_1-contact time (min)	1	5.261E+6	5.261E+6	62.09	0.0043	**
X_2-desorption time (min)	1	82464.69	82464.69	0.9732	0.3966	
X_1X_2	1	6.098E+7	6.098E+7	719.60	0.0001	***
X_1^2	1	1.184E+7	1.184E+7	78.0952	0.003054	**
X_2^2	1	2.115E+7	2.115E+7	249.59	0.00055	***
**Residual**	3	2.542E+5	84738.85			
Lack of Fit	1	17676.46	17676.46	0.1495	0.7363	Not significant
Pure Error	2	2.365E+5	1.183E+5			

$R^2 = 0.9973$, Adj-$R^2 = 0.9928$, Pred-$R^2 = 0.9876$, CV = 3.17%, Adeq-Precision = 54.9865

* $p < 0.05$
** $p < 0.01$
*** $p < 0.001$

Table 4. ANOVA results and statistical validation parameters for estriol (E3).

**Source**	**df**	**Sum of Squares**	**Mean Square**	***F-value***	***p-value***	**Significant**
**Model**	5	1.589E+9	3.197E+8	38.33	0.0064	**
X_1-contact time (min)	1	8.043E+6	8.043E+6	0.9644	0.3985	
X_2-desorption time (min)	1	1.007E+9	1.007E+9	120.79	0.0016	**
X_1X_2	1	2.765E+8	2.765E+8	33.16	0.0104	*
X_1^2	1	9.066E+7	9.066E+7	18.04	0.02390	*
X_2^2	1	1.559E+8	1.559E+8	18.70	0.02280	*
**Residual**	3	2.502E+7	8.339E+6			
Lack of Fit	1	7.742E+6	7.742E+6	0.8962	0.4437	Not significant
Pure Error	2	1.728E+7	8.638E+6			

$R^2 = 0.9846$, Adj-$R^2 = 0.9589$, Pred-$R^2 = 0.8044$, CV = 8.78%, Adeq-Precision = 29.0098
* $p < 0.05$
** $p < 0.01$
*** $p < 0.001$

AGREEprep

Analytical Greenness Metric
for Sample Preparation

17/03/2026 09:54:26

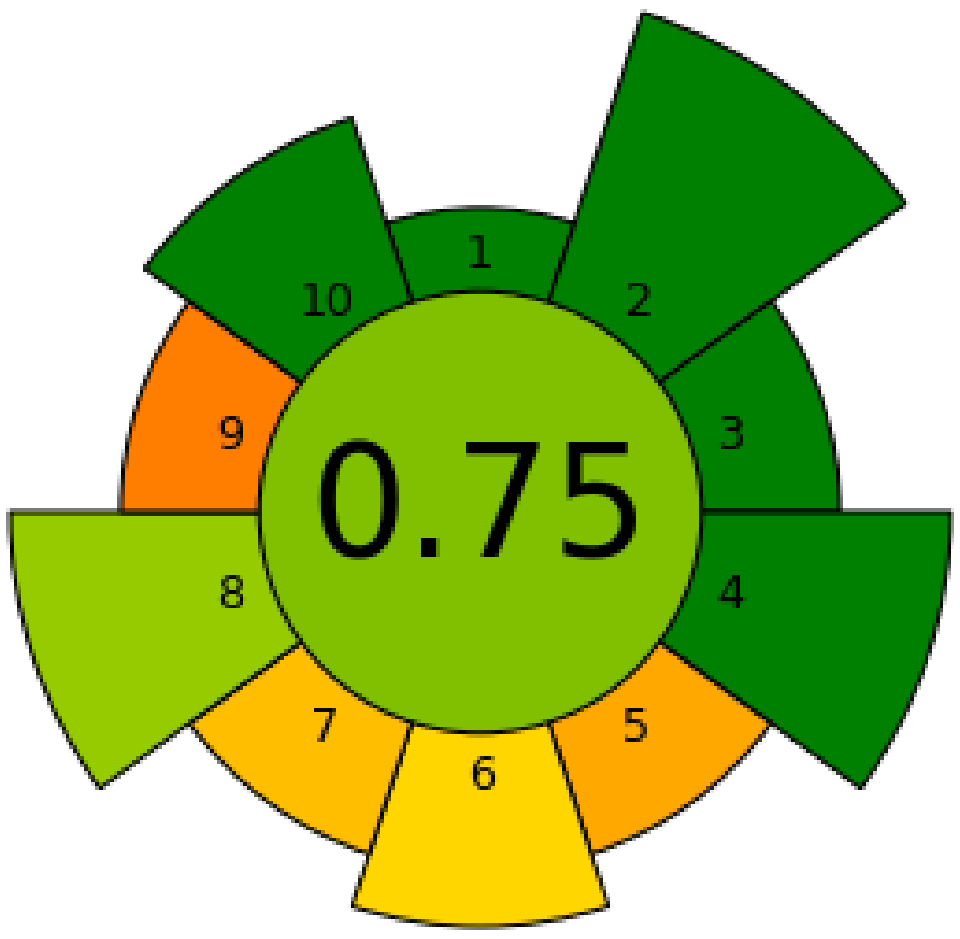

#	Criterion	Score	Weight
1.	**Sample preparation placement**<br>Sample preparation placement: In-line/In situ	1.0	1
2.	**Hazardous materials**<br>Mass [g] or volume [mL] of problematic materials: 0	1.0	5
3.	**Sustainability, renewability, and reusability of materials**<br>Only sustainable and renewable materials are used SEVERAL TIMES	1.0	2
4.	**Waste**<br>Mass [g] or volume [mL] of waste: 0.01	1.0	4
5.	**Size economy of the sample**<br>Mass [g] or volume [mL] of the sample: 10	0.33	2
6.	**Sample throughput**<br>Hourly sample throughput: 6	0.42	3
7.	**Integration and automation**<br>No. of sample prep. steps: 3 steps; degree if automation: Semi-automated systems	0.38	2
8.	**Energy consumption**<br>Approximate energy consumption per analysis [W]: 31	0.71	4
9.	**Post-sample preparation configuration for analysis**<br>Liquid chromatography, gas chromatography with quadrupole detection, etc.	0.25	2
10.	**Operator's safety**<br>No. of distinct hazards: No hazards or no exposure	1.0	3

Table S3. Sustainability comparison of representative microextraction methods for steroid estrogen determination in environmental waters based on the systematic literature review and the proposed RDSE method.

Technique / Study	Matrix / Application	AGREEprep	BAGI	VIGI	Remarks
MEPS	Reference methodology reported by Peña-Pereira *et al.*	0.55	NR	NR	Representative case study used to validate the AGREEprep metric.
SPME	Reference methodology reported by Peña-Pereira *et al.*	0.53	NR	NR	Representative case study used to validate the AGREEprep metric.
Various microextraction techniques (systematic review)	MEPS, SPME, FPSE, SBSE, MSPE, and TFME for the determination of analytes in different matrices	0.22–0.65	Not evaluated	Not evaluated	Systematic review of microextraction techniques; only AGREEprep scores were reported.
RDSE–MMT–hydroxyapatite	Extraction of steroid estrogens from hospital wastewater	0.7	50	60	Most closely related RDSE methodology reported in the literature; included for comparison of BAGI and VIGI values.

This work (RDSE–MMT–Fungus)	Extraction of steroid estrogens from hospital wastewater using an MMT–Fungus biohybrid sorbent	0.75	52.5	80	First application integrating AGREEprep, BAGI, and VIGI to evaluate the sustainability, applicability, and innovation of an RDSE method for the determination of steroid estrogens in wastewater.

NR: Not reported.

Table S4. Physicochemical properties and chemical structures of the target estrogens

Estrogen	MW ($g\ mol^{-1}$)	Log K_ow	pKa	Structure
Estrone (E1)	270.4	3.5–4.5	10.34	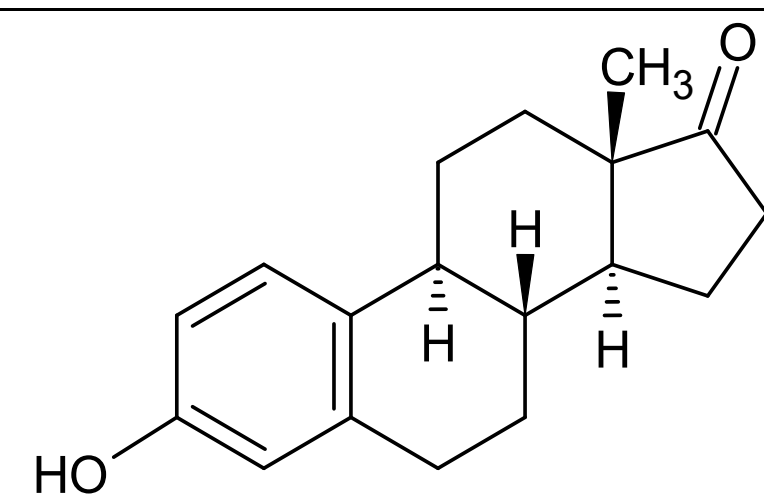
17 β-Estradiol (E2)	272.4	3.5–4.0	10.46	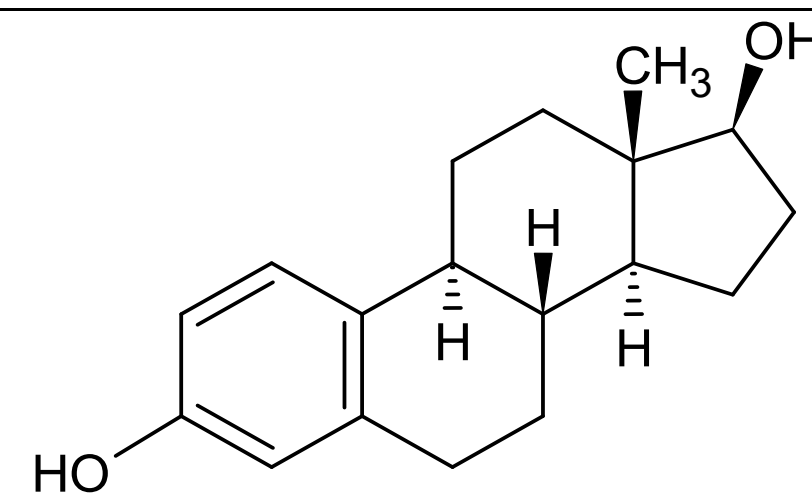
Estriol (E3)	288.4	3.0–3.5	10.54	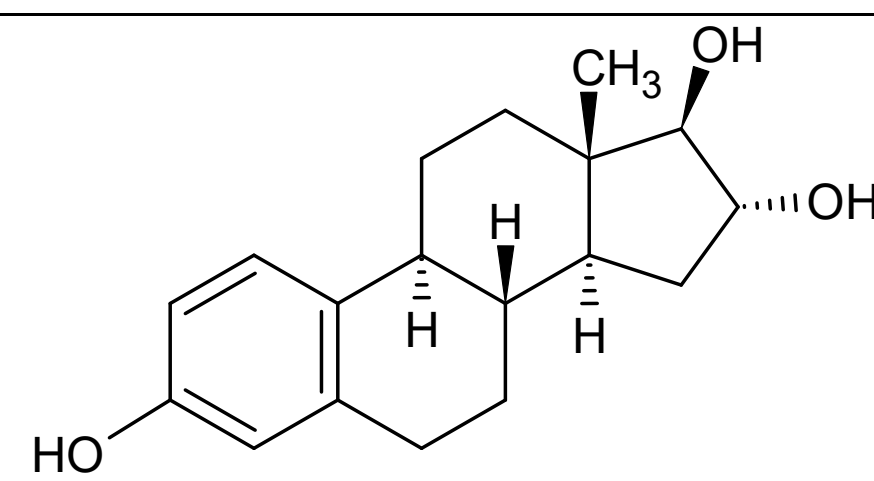
17α-ethinylestradiol (EE2)	296.4	3.5–4.5	10.33	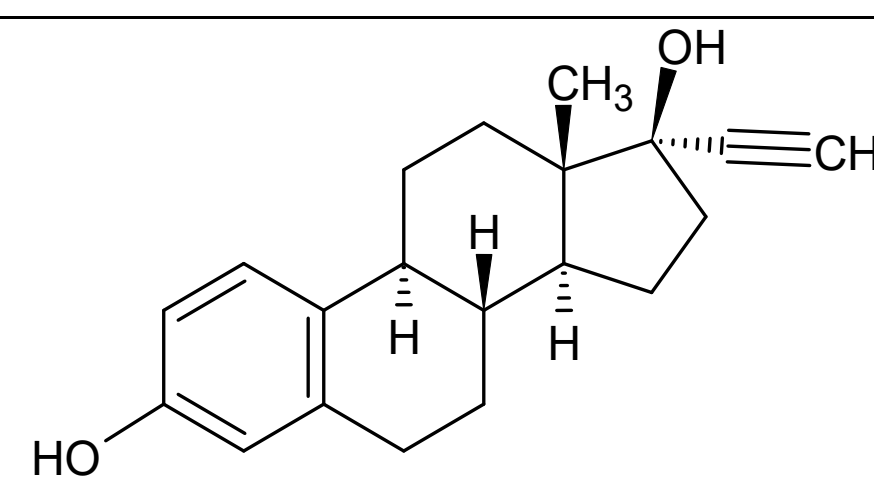

Abbreviations: MW, molecular weight; log K_ow, octanol–water partition coefficient; pK_a, acid dissociation constant.

Tables S5. Doehlert design.

Experiment	Tr: 12.639	Tr: 12.925	Tr: 13.216	Tr: 14.369
	Peak areas			
	Estrone (E1)	17β-estradiol (E2)	17α-ethinylestradiol (EE2)	Estriol (E3)
1_1	47730	687712	17834	51866
1_2	48018	770633	5842	47501
1_3	48018	770633	5842	47501
arithmetic mean	47922	742993	9839.33	48956
2_1	111221	62500	25902	16580
2_2	153156	62612	6259	16583
2_3	212606	62724	2517	16586
arithmetic mean	158994	62612	11559.3	16583
3_1	7273	1105	2505	15800
3_2	22580	4112	2565	15860
3_3	14786	4762	2623	15923
arithmetic mean	14879.7	3326.33	2564.33	15861
4_1	165945	39000	3299	12100
4_2	5980	39090	15570	12160
4_3	183439	39183	2946	12218.5
arithmetic mean	118455	39091	7271.67	12159.5
5_1	41573	11000	1998	21300
5_2	29474	11080	3440	21410
5_3	29753	11151	4276	21526
arithmetic mean	33600	11077	3238	21412
6_1	26746	1619	2069	500
6_2	19502	1833	2475	600
6_3	26657	1836	2409	667
arithmetic mean	24301.7	1762.7	2317.67	589
7_1	2018	740000	3105	6500
7_2	37265	745110	7158	6613
7_3	25430	750220	3932	6726
arithmetic mean	21571	745110	4731.67	6613
8_1	5457	4107	2700	16800
8_2	5350	3984	2781	16962
8_3	4647	4384	2862	17124
arithmetic mean	5151.33	4158.33	2781	16962
9_1	16347	1029	2159	31500
9_2	13651	6078	4896	31700
9_3	23513	15128	3335	31879
arithmetic mean	17837	7411.67	3463.33	31693

std	Experiment	Encoded values		Real values	
		X1	X2	contact time (min)	desorption time (min)
5	1	0.5	0.866	75	50
6	2	-0.5	-0.866	45	10
4	3	0	0	60	20
1	4	-1	0	30	20
2	5	0	0	60	20
3	6	0.5	-0.866	75	10
9	7	1	0	90	20
7	8	0	0	60	20
8	9	-0.5	0.866	45	50

Value	X1				
Encoded	-1	-0.5	0	0.5	1
Real	30	45	60	75	90

Value	X2		
Encoded	-0.816	0	0.816
Real	10	20	50

Tr: 12.639	Tr: 12.925	Tr: 13.216	Tr: 14.369
**Estrone (E1)**	**17β-estradiol (E2)**	**17α-ethinylestradiol (EE2)**	**Estriol (E3)**
47922	742993	9839.33	48956
158994	62612	11559.3	16583
14879.7	3326.33	2564.33	15861
118455	39091	7271.67	12159.5
33600	11077	3238	21412
24301.7	1762.7	2317.67	589
21571	745110	4731.67	6613
5151.33	4158.33	2781	16962
17837	7411.67	3463.33	31693
